# Intra‐His bundle block with Wenckebach phenomenon

**DOI:** 10.1002/joa3.12616

**Published:** 2021-08-10

**Authors:** Sharath Kumar, Garry Thomas, David Newman, Eugene Crystal, Ilan Lashevsky

**Affiliations:** ^1^ Sunnybrook Health Sciences Center University of Toronto Toronto Ontario Canada

**Keywords:** AV block, electrocardiogram, intra‐His bundle block

## Abstract

A 66‐year‐old lady presented with shortness of breath and a Wenckebach atrioventricular (AV) conduction pattern on the ECG. The electrophysiologic study showed split‐His potentials and intra‐Hisian Wenckebach. The case highlights the interesting finding of Wenckebach conduction in the His bundle.

## Graphical abstract

A 66‐year‐old lady presented with shortness of breath and a Wenckebach conduction pattern on the ECG. The EP study showed split his potentials and intrahisian Wenckebach. The case highlights the interesting finding of Wenckebach in the His bundle.

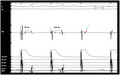

A 66‐year‐old lady with obstructive sleep apnea (OSA) presented with dyspnea. She had no syncope. The echocardiogram showed normal left ventricular function and no structural heart disease and a workup for ischemia was negative. She was not on any medications known to suppress the conduction system. Her resting electrocardiogram (ECG) showed episodes of 3:2 and 4:3 conduction with a normal QRS duration suggesting a Mobitz 1 block. (Figure 1A). The Holter revealed multiple episodes of 2:1 AV block without prolonged pauses or dysrhythmias. She underwent an exercise stress test during which she could exercise for only 2.5 min with a sinus rate of 115 beats/min and occasional nonconducted P waves (Figure 1B). An electrophysiologic study (EPS) was conducted to ascertain the site of AV block and clarify possible indications for a pacemaker. The EPS was conducted with catheters at His position and RV apex. During atrial pacing at a cycle length of 700 ms, the intracardiac electrograms showed splitting of the His potential with progressive prolongation of the intra‐His bundle split potentials culminating with a block after the first His potential, thereby indicating intra‐His bundle block (Figure 2). A permanent pacemaker was implanted in view of the intra‐Hisian block and the patient has been asymptomatic after this at 2 years of follow‐up.

**FIGURE 1:**
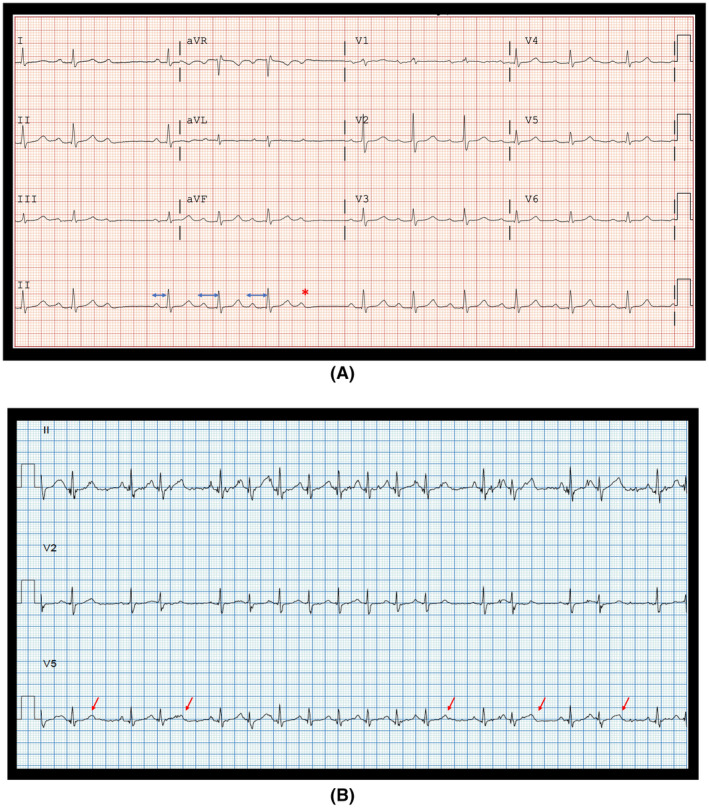
A, Baseline ECG showing 3:2 and 4:3 Wenkebach periodicity (Mobitz 1) with a narrow QRS. The arrows depict the modest increase in the PR interval with a nonconducted P wave (*). B, ECG during exercise showing occasional nonconducted P waves (shown with arrows) at a sinus rate of 115 beats/min

**FIGURE 2:**
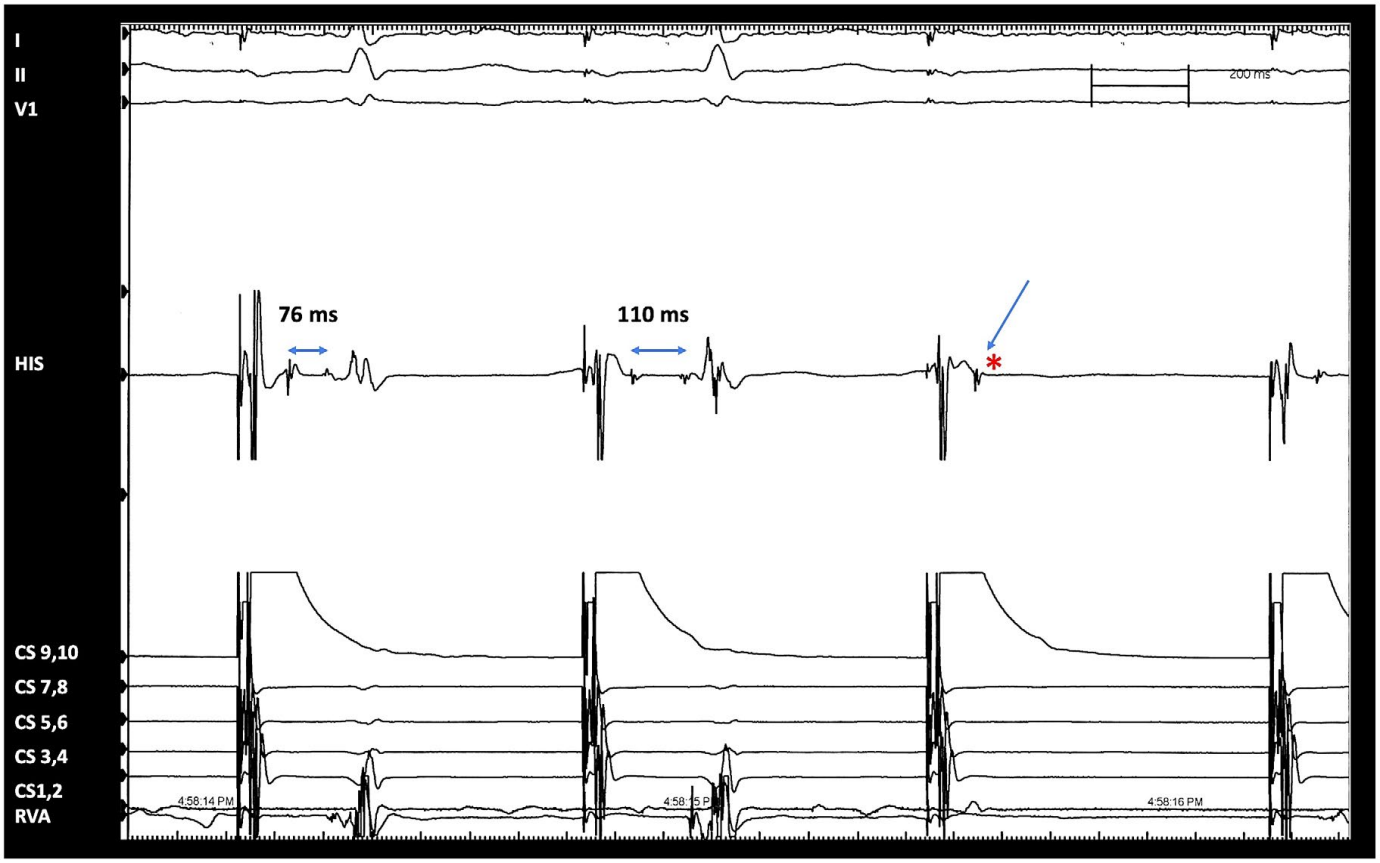
Electrophysiologic study showing split‐His potential with intra‐Hisian Wenckebach and a block within the His. The intra‐His conduction delay increases from 76 to 110 ms and culminates with a block (*) after the first His potential (marked by a vertical arrow). A right bundle potential is also evident on the RV lead

## 1 DISCUSSION

This case highlights intra‐His bundle Wenckebach presenting only with dyspnea displaying Mobitz I‐like surface ECG. Mobitz I type of AV block is generally regarded as a benign finding, but in some cases, this may indicate a more sinister problem like intra‐His bundle block. Though it may be difficult in most cases, surface ECG may offer subtle clues to the presence of intra‐His bundle block.^1^, ^2^ The presence of a normal PR interval in the setting of 2:1 block and the absence of septal q waves in the lateral leads could be indicators of intra‐His bundle block. The absence of septal Q waves may be attributed to two mechanisms: i) an infra‐Hisian escape may bypass the fibers that activate the septum and ii) bradycardia‐dependent incomplete left bundle branch block.^2^ Mobitz I block at the level of the AV node would be expected to improve with exercise and atropine. But in our case, exercise did not improve it, this is an important clue to the presence of intra‐Hisian block presenting as Mobitz 1 AV block.

Intra‐His bundle blocks with split His bundle potentials are occasionally observed in practice with intra‐His bundle Wenckebach phenomenon being rare. The largest clinical series on intra‐His bundle blocks by Guimond and Puech showed that the majority of the patients were elderly women and they presented as third‐degree AV block with syncope as the predominant symptom.^3^ The incidence of intra‐His bundle block causing heart block ranges from 15% to 27% in different studies. This type of AV block has been observed with uncommon etiologies like Rheumatoid arthritis, arteritis, and mitral annular calcification.^4^ The etiology of the conduction disturbance in our case is probably degenerative, but an association with OSA is conceivable. Intra‐His bundle block has been associated with underlying heart disease in 43% and long‐term mortality of 23%.^3^

Conventionally, the management of AV block has been based on the pattern of AV conduction, but our case illustrates that the site of block rather than the surface ECG pattern should dictate management and prognosis.^5^

In summary, intra‐His bundle Wenckebach is a rare mechanism of Mobitz I AV block pattern and should be considered in symptomatic patients presenting with Wenckebach pattern with a narrow QRS and normal PR interval.

## CONFLICT OF INTEREST

All the authors have no relevant conflict of interest to disclose.
